# A qualitative study of social accountability translation: from mission to living it

**DOI:** 10.1186/s12909-024-05093-y

**Published:** 2024-02-14

**Authors:** Jennifer Cleland, Anand Zachariah, Sarah David, Anna Pulimood, Amudha Poobalan

**Affiliations:** 1 Medical Education Research and Scholarship Unit, Lee Kong Chian School of Medicine, Nanyang Technological University, 11 Mandalay Road, Singapore, 308232 Singapore; 2 Christian Medical College, M.G.R Medical University, Vellore, India; 3 School of Medicine, Medical Sciences and Nutrition, University of Aberdeen, Aberdeen, UK

**Keywords:** Social accountability, Qualitative research, Under-resourced setting

## Abstract

**Background:**

Medical schools are increasingly adopting socially accountable mission and curricula, the realisation of which are dependent on engaging individuals to embody the mission’s principles in their everyday activities as doctors. However, little is known about how graduates perceive the efforts taken by their medical school to sensitise them to social accountability values, and how they translate this into their working lives. Our aim was to explore and understand graduate perceptions of how their medical school influenced them to embody a social accountability mission in their working lives.

**Methods:**

This was a qualitative interview study carried out with graduates/alumni [*n* = 51] of Christian Medical College, Vellore [CMCV], India, a school with a long-established and explicit social-accountability mission. Data coding and analysis were initially inductive and thematic using Braun and Clarke’s six step framework. MacIntyre’s virtue ethics theory framed secondary analysis, allowing us to consider the relationships between individual and contextual factors.

**Results:**

Our participants perceived that CMCV invested heavily in selecting personal qualities aligned with the CMCV mission. They saw that these qualities were reinforced through various practices: [e.g., placements in resource limited and/or remote and rural settings]; community engagement and expectations [e.g., student self-governance]; role modelling [staff and more senior students]. Much emphasis was placed on sustaining these traditions and practices over time, creating a strong sense of identity and belonging among participants, traditions which were fostered further by the alumni network and continued engagement with CMCV post-graduation.

**Conclusions:**

Ensuring social accountable medical education depends on alignment and interactions over time between context and structures, systems and human agents. Further studies are needed to extend understanding of how students from diverse contexts experience socially accountable medical education and translate their educational experience into their thinking and practice after graduation.

## Introduction

The World Health Organization [WHO] defines social accountability, in the context of medical education, as the responsibility to focus education, research, and service activities on tackling the most pressing health issues in the communities, regions, and countries they have a mandate to serve [1]. Increasingly, medical training institutions globally are using this framework to design their curricula [2–6] and prepare future doctors to be responsive to the needs of the community, region and/or nation [7, 8].

With this has come a proliferation of research examining different aspects of social accountability in medical education. Studies in this area focus on five broad areas of inquiry: description of this mission and curricula [e.g., 9–12]; guidelines for implementing and assessing social accountability curricula and mission [e.g., 6, 8, 13–15]; challenges to integrating social accountability into curricula [e.g., 16–18]; who is selected into schools with an explicit social accountable mission [e.g., 19–21] and where the graduates from these schools ultimately practice [e.g., 8, 22–24]. The few studies of student experience have focused on student perceptions of formal [planned and timetabled] mechanisms and experiences at socially accountable medical schools [e.g., 25, 26]. However, we lack understanding of how students carry the mission they encounter at medical school forward into their thinking and practice after graduation. This is problematic as without examining this space we do not have a full sense of the essential elements or what matters in terms of achieving social accountable medical education. In other words, if “the realisation of a social mission is dependent on an institution’s ability to direct or persuade individuals within it to embody the mission’s principles in their everyday activities” [25, p. 172], in-depth understanding of how learners experience the social accountability mission of their medical school as students and how they embrace this going forward is critical.

Therefore, to address this gap in knowledge, our aim was to explore and understand graduate perceptions of how their medical school influenced its students to embody a social accountability mission. Our specific interest is in graduates/alumni perspectives of their time at medical school and how they carried that experience with them after graduation. Graduate narratives are built in the present but are formulated and sorted based on the application of experience [27]. They have had time to put their education and career in perspective [28] and thus provide unique insight. Indeed, graduate/alumni research has been deemed important in respect of informing curriculum development and assessing learning outcomes [e.g., 29], but their voices are rarely heard in health professions education research.

## Methods

This was a qualitative interview study underpinned by social constructionism, acknowledging that reality is produced through interchanges between people and objects and shared activities, with knowledge and the individual embedded within history, context, culture, language and experience [30].

### Setting

Our context was a medical school which has had an explicit social accountability mission for over 100 years. Christian Medical College, Vellore, Tamil Nadu, India, [CMCV] was established as a not-for-profit medical school for women in 1918 [men were admitted from 1947] with the explicit principles of preparing graduates to meet health care needs of society and with social values at its core. Much teaching and learning is delivered in the community, in rural, tribal, urban and in urban slums and CMCV has links with 150 secondary mission hospitals across India. CMCV supports the mission hospitals via the CMCV graduate service obligation: students selected from local communities go back and work in these communities for a two-year service obligation. However, there is the expectation that all CMCV graduates will choose to work in areas of need and deprivation, whether these be rural or urban.

### Data collection

We had three orientation discussions with senior faculty [via Zoom], the CMCV website and a site visit [November 2019] to inform the research focus. During this orientation, JC used her outsider [“etic”: 31] status to ask questions about the history and traditions of medical education in India generally and CMCV’s activities and processes specifically. We used this information and the social accountability literature as the basis for developing a semi-structured interview schedule. Questions included: where alumni worked [the health care provision of their hospital/organisation], why they chose this work, and their perceptions of the relationship between their medical education experiences and their practice/career choices. We tested and refined our questions via two pilot interviews. No substantial changes were required to the schedule following piloting, so we incorporated these data into the main data set.

### Sampling and recruitment

We wanted to explore how CMCV created an environment that supported students to integrate their personal beliefs and motivations with CMCV’s social accountability mandate. To gather a range of perspectives, we aimed to recruit CMCV graduates/alumni from a wide variety of backgrounds, working in different regions and countries and across diverse healthcare settings.

CMCV’s Graduate Office and Alumni Directorate records mapped out details of CMCV graduates working in India, by geographic location [including urban, rural, slums], type of health work [hospital-based work, community health work, inter-sectoral involvement] and different sectors [mission/charity/government], over 50 years [graduates from class of 1960s to 2010]. In addition, we generated a list of CMCV graduates living abroad. From this list, we purposively approached respondents who represented a range of sexes, specialties, type of work and geographical spread [32]. Demographic and career data were cross-referenced via an accompanying short questionnaire prior to the interviews.

In January 2020, the CMCV Alumni office emailed invitation letters to take part in the study, the participant information sheet [PIS] and consent form to graduates. They sent a reminder after two weeks. We followed up on indications of interest in the study by arranging a face-to-face or remote interview. Formal consent was obtained before data collection. At this point, we also reminded participants that their data would be held securely and anonymized for reporting, and they could withdraw at any point during or after the interviews without explanation. As far as possible, open questions guided the discussions, with prompts from the researcher [SD] to probe for deeper understanding of participants’ views. All interviews were undertaken by SD. The interview schedule ensured consistency, but interviews were iterative. Data were collected in English between January and December 2020.

### Data management and analysis

Participant interviews were iterative and digitally audio-recorded, transcribed verbatim by a third party approved by CMCV, and anonymised during transcription. In keeping with our constructivist stance, initial data analysis was inductive, thematic and reflexive [33, 34]. SD and AP carried out the initial analysis, using Braun and Clarke’s six step framework to generate themes while constantly reflecting on their relationship with the data. JC then examined the data and SD/AP’s interpretation thereof, while constantly considering her own positionality [see later for further discussion] [31]. AP and JC had regular meetings to reflect on the data and consider how best to represent meaning as communicated by the participants. The resultant codes were then shared with the wider team for further discussion and reflection. Ideas were documented through memos and correspondence that created an audit trail of the analytical process.

In keeping with CMCV’s ethos of collaboration and engagement with its alumni [35, and see Results], we also invited some of the research participants to check a draft manuscript outlining our initial interpretations [36, 37], asking them if they could see their experiences within the results and if they wanted to add anything for us to consider [38]. Eight interviewees came back to us with feedback and comments, and we analysed these as part of the final data set.

During the inductive analysis we were struck by our participants’ references to enacting the social accountability mission of CMCV, not just in respect of where they went onto work, but how they acted within the world. Given this, we then used MacIntyre’s virtue ethics theory as a framework within which our data could be analysed. Drawing from Aristotle’s work on ethics, the contemporary philosopher Alasdair MacIntyre [39, 40] developed a “virtues–practices–goods-institutions” framework where virtue is dispositional (qualities of character) and guides actions, but these dispositional qualities can be reinforced and sustained (or not) by the social, cultural and political context within which an individual operates. For example, a student’s intrinsic commitment to the social accountability mission espoused by the school may be reinforced (or not) through the curricular and other experiences offered, and this combination of disposition and reinforcement will inform career choices. The use of this framework thus enabled us to consider the relationships between character (disposition) and CMC (the institution, its traditions and structure), and how CMC supported the processes of learning to be virtuous in respect of social accountability. MacIntyre’s virtual ethics theory has been used extensively in sociology and organizational research [see, for example, 41–43 for overviews], more recently in medicine [e.g., 44] and medical education [45].

### Rigour and reflexivity

We reflected on our backgrounds and how these may have shaped our interpretation of the data. Our identities differ in terms of ethnicity, gender, religious orientation, learning experiences and disciplinary backgrounds, research interests and personal life courses [46, 47]. We are based in three countries, each of which represents very different contexts in terms of power and privilege, and how access and opportunity are distributed in society [48, 49]. We also constantly considered our insider [AP [a CMCV graduate working in the UK], APul, AZ, SD] and outsider status [JC] [31]. Team discussions were thoughtful, respecting our different views and positions in relation to the data.

### Patient and public involvement

Patients and the public were not involved in study planning.

### Ethics

Ethical approval was obtained from the Institutional Review Board [IRB] of CMC, India [IRB 12,141] and College Ethics Review Board [CERB], University of Aberdeen [CERB/2019/8/1819].

## Results

We conducted 51 interviews with a total of 54 participants: 48 individual interviews [M = 32; F = 16] and three with two graduates in each interview [medical couples who had both graduated from CMCV]. Four participants were graduates from the 1950-60 decade; 14 from 1960 to 70; 19 from 1970 to 80; 12 from 1980 to 90, two from 1990 to 2000 and three participants were graduates from 2000 onwards. While many participants had worked in diverse sectors and settings over their careers, at the time of data collection, twenty-eight interviewees were working in Mission hospitals or NGOs [non-governmental, non-profit organizations]; five in the Private/ Corporate sector and others in the Government sector. Most worked in India: 12 participants worked abroad. Half of the participants [*n* = 27] worked in rural or semi-urban areas. Interviews lasted for an average for 31 min ranging from 22 to 41 min.

We start with themes and data relating to CMCV structures: from selection, through to remote and rural placements and post-graduate service. We then present data relating to traditions and narratives around these traditions, role modelling and community expectations. Finally, we report data on “action”, on participants’ perceptions of how their CMCV training influenced their career decisions and other actions, and how they contribute to sustaining CMCV’s mission.

Quotations are included to aid confirmability and to help the reader follow the logic of the story. Participants are labelled by gender, place of work [urban/rural], type of workplace [e.g., Government hospital] and time of joining CMCV [e.g., class of 60–70].

### Selecting for certain personal qualities

The orienting interviews and webpages illuminated that CMCV selected for the character qualities or values they considered necessary to work in healthcare [e.g., compassion, empathy, respect, service to the community] rather than assuming these qualities could be developed during medical education and training. To achieve this, while academic attainment and aptitude tests were used as the first stages of selection, the final stage of the selection process was a three-day selection centre mapped to CMCV graduate outcomes [“backwards chaining”, 50]. This selection centre was designed to allow candidates multiple situations to demonstrate key skills, personal attributes and values. During the selection centre, applicants resided on campus and had much contact with existing students and staff. Over time this intensive and resource-costly process was modified but the basic steps and the core guiding principles remained intact [51, 52]:

“I saw the system, and the way they treated me and the group observers [during interviews], how…you know, how much value they give to human values.” [Male, Urban, Corporate Hospital, Class of 80-90s].

The CMCV selection process seemed to be both a structure and tradition, a way of communicating and sustaining CMCV’s values to potential applicants and other stakeholders:

“So, it was very clear that our institution seemed to attract people who had this particular calling to serve others.” [Male, Retired, CMC Medical Education, class of 60-70s].

### Reinforcing inherent personal qualities through practices

#### Curriculum

Values and commitment traits identified during the selection were nurtured and shaped through all aspect of the curriculum [53]. For example, formal training was aligned with the needs of the communities CMCV serves [resource limited and/or remote and rural settings] and the focus was on preparing the students for those specific working environments in remote and rural communities via early clinical exposure, building clinical skills, and taking on increasing responsibility for patient care:

“We went to these mission hospitals where we were able to see actually what medical practice is in the rural areas where there is dearth of technology, …where we need to be very cost effective and conscious of the resources…. All our teaching and our learning was sort of geared in that direction” [Female, Mission hospital, Rural, class of 80-90s].

### Community engagement and expectations

There was a tradition of expecting students to take on social and administrative responsibilities associated with campus living and social service activities [e.g., delivering healthcare of disadvantaged people in the area]. This was designed to help students reach their potential [e.g., in teamworking and leadership skills] and encourage students to actively contribute to the community, thus drawing students into the CMCV community:

“ [Roles in the Hostel Union provide] the opportunity to create budgets- to execute them… gained some experience and knowledge, through all those small post… that we took, helped us later in running mission hospitals” [Male, Mission hospital, Rural, class of 80-90s].

There were also close connections between students and faculty. This was encouraged via a formal programme where students were linked to campus faculty through foster families. However, there were also many social and informal opportunities to mix with and to learn from faculty which helped foster community life:

“It was very personalized. We knew our teachers. We were able to relate to them. We could tell them things. They understood us. They knew us by name. They called us to their homes.” [Male, Mission hospital, Urban, class of 60-70s].

“…I mean there was no kind of a barrier to approach a faculty for any help, be it in studies related or your personal life, wouldn’t matter.” [Male, Urban, Abroad, class 2000-10s].

As mentioned earlier, students were selected for their values in respect of social accountability and the data makes clear that the CMCV values were rooted in social responsibility. Although a predominantly Christian college, students came from different backgrounds and acceptance of difference was encouraged:

“So, we had lots of debates, lots of debates, because we were living together, and so they’d be lot of things, not only about the Bible, also about public work, about what we will go and do ….” [Male, NGO, Rural, class of 70-80s].

### Role modelling - seeing others “live it” and by “living it”

Our participants thought the values-driven formal curriculum was enhanced by the role modelling provided by teachers, seniors and mentors. Faculty seemed to inculcate the value of compassion and attending to the needs of others [the students] through their interest and commitment to supporting students, as well as their own medical, academic and personal practices [e.g., opting to deliver service rather than seek high status jobs or salaries elsewhere]. This made a long-lasting impact on many participants who spoke of the role modelling they experienced, reflecting the school’s strong culture of ethical living and patient care at the centre:

“I think that having seen many of my faculty and teachers, the way they handle people, relationships and the importance of maintaining good relationship with people was very helpful… to really value every person” [Male, Mission Hospital Admin, Semiurban, class of 80-90s].

“We could see our seniors especially in the hostel association administration as well as the student’s union how matured and responsible they were and we were able to emulate them as we grew” [Male, Mission hospital, Semiurban, class of 60-70s].

This role modelling seemed to help them internalize CMCV’s values and behave accordingly.

### Sustaining practices

#### Sustaining the traditions

The communal living with personal, economic and religious diversity, exposure to remote and rural communities early in the training, strong work ethic and taking responsibility for the care and wellbeing of the CMCV community as well as their patients, created a strong sense of group identity and belonging among participants. They discussed how these experiences influenced them, and differentiated them from other medical school graduates in terms of public service and practicing care in the community:

“You should not, you know, use shortcuts to make a quick buck. The things that I took for granted there [CMCV] which when I came here [workplace now], I remember somebody telling me that, you know, what sets you apart is your very strong values that you hold on to” [Female, Private, Urban, class of 60-70s].

In other words, participants described themselves as part of a recognizable group with a clear purpose that differentiated them from graduates from other schools.

### Supporting the community [CMCV]

The alumni records from which we drew our sample suggested that a high proportion of CMCV graduates were working in [or had worked in] mission hospitals or NGOs, and/or in rural or semi-urban localities, and drew on what they had learned at CMCV to inform their career and practice decisions. There was a strong sense of bond with the institution which seemed to help CMCV alumni to sustain lifelong learning and get help when working in resource limited settings:

“It is like you become a part of the family of CMC and you never leave… we can always run to this haven of learning and receive so much.” [Female, Mission hospital, Rural, class of 80-90s].

Related to this was the role of the alumni association in maintaining the linkage between the college and its alumni. The alumni association sustained the sense of continuing belonging of the alumni to CMCV through its many activities [e.g., fund raising, reunions]. There was also a tradition of a high level of involvement of alumni within current students [for example, alumni speak to the students about their work, collaborate on teaching and research, and many alumni come back to engage in collaborations and/or work at CMCV], and these alumni communicate and sustain practices; they are a means by which students are educated into CMCV’s practices.

## Discussion

### Principal findings

In summary, CMCV had constructed a coherence of culture, structures and processes to pass on their values and traditions to, and through, students who had been selected because they had an inherent disposition towards socially accountable service. During their time in the CMCV community students learned both socially accountable ways of working and a position from which to act in the world. Then, by enacting CMCV virtues in their careers and engaging with CMCV as alumni, CMCV graduates sustained the traditions which provided both practices and individual lives with value.

### Strengths and weaknesses of the study

Our study is carried out in the context of one medical school in one country so we cannot assume our findings are generalizable to other contexts. However, the messages from the study are pertinent to all medical schools with a social accountability mission. Our engagement with graduates rather than current students or staff adds to the perspectives already explored in the body of literature on this topic [e.g., 25, 26]. This gave insight into participants’ experiences of a socially accountable education and their views of this post-graduation. Of course, data collection inherently depended on participant recall, but the data suggested that our participants had very clear memories of their time at CMCV. Moreover, the retrospective interview is an accepted method of knowledge construction which can contribute to the understanding of processes in educational practice [54, 55].

We do not know if the views and career actions of our interviewees are typical of all CMCV graduates. It may be that those alumni who engaged with the research are those who positively embraced the social accountability values of the institution. There may be other CMCV alumni who did not do so. A large-scale survey of CMCV graduates would be useful, to gather more data on their values and career choices. This data could then be compared with that of graduates from other medical schools set up with social accountability missions elsewhere in India and in other countries. We also suggest the need for future qualitative work comparing the views of alumni from different medical schools, and how they have embraced the social accountability mission of their medical school into their work, would be useful, to discover if what we found is unique to CMC or more widespread. Most of our interviewees had graduated before 2000: more recent graduates may have different views. We have no way of knowing but we suggest that this would be the case in any research of this nature. Future studies may wish to adopt approaches which highlight differences in experience over time and how such differences influence attitudes and work choices.

We used MacIntyre’s theory with its dual emphasis on context, practices and structures, and human characteristics, to aid conceptual generalisability [transferability], not to judge or defend CMCV practices. This theory has its detractors [e.g., 56] and obviously, any one theory only illuminates certain aspects of the data [57]. However, like others [41–45], we found it useful for organising our findings to make clear what was seen as important in terms of developing social accountability by our participants.

### Unanswered questions and future research

Students and graduates, the care providers, are only one part of the jigsaw puzzle of social accountability. We suggest that future work examining the translation of social accountable missions to action and future research may wish to engage with patients as well as health care providers and educators.

Our data suggests that social accountability within medical education should be viewed as holistic and complex, dependent on alignment and interactions over time between context and structures, systems and human agents. This opens the door to future research using in-depth qualitative approaches. For example, case study methodology [58] would enable further, detailed explorations of the relationships and systems related to social accountability within other “tidal pools” [that is, medical schools] and their contexts at different points in time and over time [59].

Third, socially accountable medical education is an area of much research activity but little use of theory in that research [60]. We suggest that if “determining whether or not progress is being made in an area of study requires judging …. whether or not the focus of our research efforts continue to evolve” [61, p. 295], our study provides an example of how theory can be used to aid transferability.

### Implications

In medical education, while acknowledging the reach of the Training for Health Equity Network [THEnet], most published reports of social accountability processes and impacts are from medical schools in high income countries. This reflects general publishing patterns in the field [e.g, 62]. In contrast, our study is from a school located in a low/middle income country [LMIC], India, which has long struggled to attract and retain doctors and healthcare professionals to remote, rural and deprived areas. This paper thus brings diversity and practices from a non-Western setting into health professions research and scholarship [63]. At the same time, our specific research question is relevant to an international audience – the focus on social accountability in health professions education is widespread even if the specific focus of this study was firmly grounded in one school, in one country.

## Conclusion

In conclusion, we suggest that the process of engaging students to embody a social accountability mission in their working lives depends on multiple, coherent and interrelated approaches and actions, and the intersection between systems and individuals. We propose that assessing the success of socially accountable medical education is as much about understanding how graduates perceive their education and how that experience influenced how they act within the world, as it is surveying where they work. This information about the reflection and enactment of social accountability can then inform the development of social accountability practices in the future.

## Data Availability

The datasets used and/or analysed during the current study available from the corresponding author on reasonable request.

## Declarations

### Transparency statement

We confirm that this manuscript is an honest, accurate, and transparent account of the study being reported; that no important aspects of the study have been omitted; and that any discrepancies from the study as planned have been explained.
